# Photosynthetic Pigments Changes of Three Phenotypes of Picocyanobacteria *Synechococcus* sp. under Different Light and Temperature Conditions

**DOI:** 10.3390/cells9092030

**Published:** 2020-09-03

**Authors:** Sylwia Śliwińska-Wilczewska, Zofia Konarzewska, Kinga Wiśniewska, Marta Konik

**Affiliations:** 1 Division of Marine Ecosystems Functioning, Institute of Oceanography, University of Gdansk, Avenue Piłsudskiego 46, P-81-378 Gdynia, Poland; zofia.konarzewska@gmail.com; 2 Division of Marine Chemistry and Environmental Protection, Institute of Oceanography, University of Gdansk, Avenue Piłsudskiego 46, P-81-378 Gdynia, Poland; kinga.wisniewska@phdstud.ug.edu.pl; 3 Department of Marine Physics, Institute of Oceanology Polish Academy of Sciences, P-81-779 Sopot, Poland; mk@iopan.gda.pl

**Keywords:** abiotic stressors, environmental stress, growth, light intensity, photosynthetic pigments, picocyanobacteria, plant physiology

## Abstract

It is estimated that the genus *Synechococcus* is responsible for about 17% of net primary production in the Global Ocean. Blooms of these organisms are observed in tropical, subtropical and even temperate zones, and they have been recorded recently even beyond the polar circle. The long-term scenarios forecast a growing expansion of *Synechococcus* sp. and its area of dominance. This is, among others, due to their high physiological plasticity in relation to changing environmental conditions. Three phenotypes of the genus *Synechococcus* sp. (Type 1, Type 2, and Type 3a) were tested in controlled laboratory conditions in order to identify their response to various irradiance (10, 55, 100 and 145 µmol photons m^−2^ s^−1^) and temperature (15, 22.5 and 30 °C) conditions. The highest total pigment content per cell was recorded at 10 μmol photons m^−2^ s^−1^ at all temperature variants with the clear dominance of phycobilins among all the pigments. In almost every variant the highest growth rate was recorded for the Type 1. The lowest growth rates were observed, in general, for the Type 3a. However, it was recognized to be less temperature sensitive in comparison to the other two types and rather light-driven with the highest plasticity and adaptation potential. The highest amounts of carotenoids were produced by Type 2 which also showed signs of the cell stress even around 55 μmol photons m^−2^ s^−1^ at 15 °C and 22.5 °C. This may imply that the Type 2 is the most susceptible to higher irradiances. Picocyanobacteria *Synechococcus* sp. require less light intensity to achieve the maximum rate of photosynthesis than larger algae. They also tolerate a wide range of temperatures which combined together make them gain a powerful competitive advantage. Our results will provide key information for the ecohydrodynamical model development. Thus, this work would be an important link in forecasting future changes in the occurrence of these organisms in the context of global warming.

## 1. Introduction

The discovery of autotrophic picoplankton in the late 1970s [1,2] has contributed to numerous scientific studies on these organisms and demonstrated their significant role as a missing link in the carbon cycle and a major producer in oceanic waters [3]. Many researchers proved that picoplankton also plays an important role in more productive waters, often exceeding the abundance and biomass of other phytoplankton species [4]. The genus *Synechococcus* is a polyphyletic group of picoplanktonic cyanobacteria that constitutes one of the major contributors to oceanic primary production [5,6] and is a key worldwide distributed component of marine planktonic communities [7]. It is estimated that for about 17% of net primary production in the Global Ocean is responsible solely the genus *Synechococcus* [8]. Blooms of these organisms are observed in tropical, subtropical and even temperate zones [9]. The present global warming causes temperature rise which was recognized as a main cause of the massive shift of species northwards [10]. Furthermore, *Synechococcus* has been recorded far beyond the polar circle, e.g., dragged with a strong Atlantic inflow in 2014, as far as 82.5° N [11]. In the future ocean scenarios, a growing expansion of *Synechococcus* sp. and its area of dominance is forecasted [8,12]. A significant increase in the frequency of their blooms has already been detected [9]. This is, among others, due to their high physiological plasticity in relation to changing environmental conditions [13]. Organisms from the genus *Synechococcus* are represented by three phenotypes that complement each other and fill tightly the ecological niche due to varying photosynthetic pigment profiles and high chromatic adaptation potential.

The photosynthetic pigment observed in cells of picoplanktonic cyanobacteria is chlorophyll *a* (Chl *a*), carotenoid (Car) pigments, and phycobiliproteins (Phyco) [14]. Chl *a* is the most important pigment because it controls photosynthesis and this transformation of the absorbed energy from sunlight into chemical compounds determines the biomass growth rates [14]. The most dominant Car pigment is zeaxanthin (Zea), representing 40% to 80%. The presence of cell-specific Zea content in *Synechococcus* sp. and high Zea/Chl *a* ratios may be regarded as a diagnostic feature [15]. Besides Zea, *β*-carotene (*β*-Car) is also present among Car pigments [16]. Car pigments play an important photoprotective role against damage to the photosystem [17]. Furthermore, cells of picocyanobacteria contain accessory phycobilin pigments instead of the additional chlorophylls that are common among other phytoplankton organisms. There are three types of Phyco containing: phycoerythrin (PE), phycocyanin (PC), and allophycocyanin (APC), which absorb green, yellow-orange, and red light, respectively [18]. In cyanobacterial cells, Phyco are organized into aggregates consisting of many subunits called phycobilisomes, which are connected in regular rows to the surface of thylakoid membranes. The main component of the core complex is APC while PE is located in the peripheral parts of these formations [19]. Phyco absorb light in the 500−650 nm range and provide additional energy to photosynthetic centers. The transfer process is highly efficient and reaches 80−90% of the energy absorbed by phycobilin pigments. Their role is vital, especially in case of any light shortages to maintain high photosynthesis rate which guarantees cyanobacteria competitive advantage in low-light conditions. The red PE absorbs the blue-green light that penetrates the deepest into the water column. It enables conducting photosynthesis even at the bottom of the euphotic zone. The deeper live an organism, the more PE it contains and the higher is the PE to Chl *a* pigment ratio. In the cells of cyanobacteria living in the upper layers of the ocean the dominant pigments are the blue PC and APC [19]. 

The distinction between the three main identified phenotypes of the genus *Synechococcus* is based on the phycobilin pigments composition [20,21]. Six et al. [22] in their research presented a classification that divides marine *Synechococcus* to Type 1, Type 2, and Type 3. Organisms with the dominance of PC were classified as Type 1. Type 2 incorporates phenotypes with a dominance of PE, more specifically PEI, while Type 3 consists of organisms in which PC, as well as PEI and PEII, dominates in phycobilisomes. Furthermore, Six et al., [22] divided Type 3 into four subcategories from a to d, according to the increasing phycoerythrobilin (PEB) and phycocyanobilin (PCB) ratios. Organisms with high levels of PE are found mainly in oligotrophic oceans, while green (PC-rich) phenotypes prefer turbid freshwater [23,24]. In general, picocyanobacteria prefer lower irradiance intensity to reach the maximum rate of photosynthesis than larger algae [25]. Furthermore, studies have shown that the reduction of radiation intensity does not change the efficiency of carbon incorporation during photosynthesis, as is the case with larger plant organisms that exceed 3 μm. Marine *Synechococcus* sp. is able to saturate photosynthesis and growth rates at very low radiation [26]. Under culture conditions, some strains of picoplankton have shown the ability to survive and grow again after periods of total darkness [27,28]. Platt et al. [29] observed photosynthetic picoplankton at a depth of 1000 m in the depths of the eastern Pacific Ocean and Cai et al. [30] confirmed the presence of small populations of *Synechococcus* sp. in the Chesapeake Bay during winter months. Furthermore, Ernst [31] isolated *Synechocystis* sp. (Maple BO 8402) from the Lake Constance with a different type of pigmentation than any described so far. This strain contained Phyco similar to the PC, characterized by very strong red fluorescence occurring after stimulation of the cells with wavelengths of 600 nm but also with wavelengths of 436 and 546 nm [32]. Most cyanobacteria, especially those living all year round in coastal ocean waters, contain PE [23,33,34].

The main aim of this study was to determine the acclimatization capacity of three Baltic phenotypes of *Synechococcus* sp.: Type 1, Type 2, Type 3a. Furthermore, the study focused on the effect of irradiance, temperature, and their mutual interactions on the content and proportions of cell-specific photosynthetic pigments of the examined cyanobacterial phenotypes. The cell-specific Chl *a* and Car content was determined by the HPLC method, whereas the content of Phyco was determined by the spectrophotometric method. The detailed characterization of the quantitative and qualitative composition of pigments is important to determine the level of acclimatization of the examined phenotypes of cyanobacteria to specific environmental conditions. The knowledge of biology and especially the physiology of these organisms by capturing their reactions to various environmental factors is important for forecasting the possible expansion of these organisms. 

## 2. Results

### 2.1. The Cell Concentration and the Growth Rate of Three Synechococcus sp. Phenotypes under Different Culture Conditions

**Figure 1:**
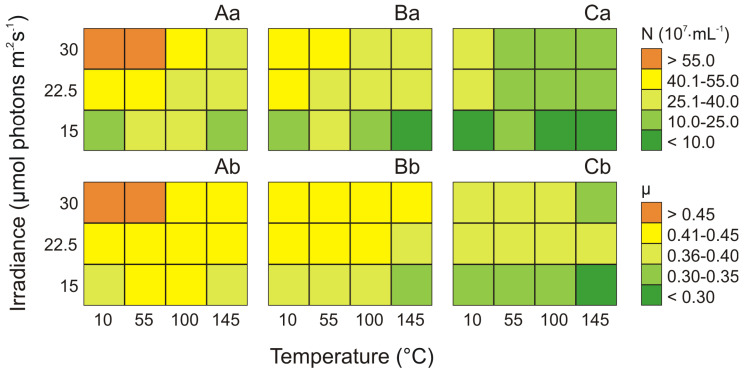
Changes in the number of cells (N × 10^7^ mL^−1^; **a**) and the growth rate (μ; **b**) obtained after 14 days of experiment for three phenotypes of *Synechococcus* sp.: Type 1 (**A**), Type 2 (**B**), Type 3a (**C**) under different irradiance (μmol photons m^−2^ s^−1^) and temperature (°C) conditions.

In this study, the concentration of picocyanobacterial cells as well as the growth rate under different irradiance and temperature conditions were determined for the three studied phenotypes of *Synechococcus* sp. (Type 1, Type 2, and Type 3a). In general, factorial tests showed that both irradiance and temperature significantly affected the number of cells of three *Synechococcus* sp. phenotypes (ANOVA, *p* < 0.001, *p* < 0.01, *p* < 0.01, for Type 1, Type 2, and Type 3a, respectively; Table S1). Moreover, ANOVA results indicated that for each picocyanobacteria phenotype the effect of temperature on the culture concentration was higher than the influence of irradiance and the interaction of both factors (Table S1). The highest picocyanobacterial cell numbers (59.5 × 10^7^ and 60.2 × 10^7^ cell mL^−1^) was noted for *Synechococcus* sp. Type 1 at 10 μmol photons m^−2^ s^−1^ and 55 μmol photons m^−2^ s^−1^, respectively and 30 °C (Figure 1Aa), and it was about 4-fold higher that the minimum values observed in 15 °C and 145 μmol photons m^−2^ s^−1^ (15.2 × 10^7^ cell mL^−1^). For *Synechococcus* sp. Type 2 (Figure 1Ba) and Type 3a (Figure 1Ca) the maximum cell concentration were recorded at the temperature of 22.5 °C and 30 °C, respectively. Moreover, the highest picocyanobacterial cell numbers for Type 2 was found at irradiance 55 μmol photons m^−2^ s^−1^ (49.4 × 10^7^ cell mL^−1^), whereas for Type 3a at 10 μmol photons m^−2^ s^−1^ (25.8 × 10^7^ cell mL^−1^). For both phenotypes, similar to Type 1, the minimum number of cells were obtained at 15 °C and 145 μmol photons m^−2^ s^−1^ (about 9.7 × 10^7^ and 6.5 × 10^7^ cell mL^−1^, respectively).

It was found that analyzed phenotypes of *Synechococcus* sp. showed different growth rates (μ) under different temperature and light conditions. For *Synechococcus* sp. Type 1, Type 2, and Type 3a the highest growth rate was recorded at the highest temperature (30 °C). Moreover, the highest growth rate for Type 1 (Figure 1Ab) and Type 2 (Figure 1Bb) was noted at 55 μmol photons m^−2^ s^−1^ (0.457, 0.443, respectively) whereas for Type 3a at 10 μmol photons m^−2^ s^−1^ (0.396; Figure 1Cb). On the other hand, for Type 1, Type 2, and Type 3a, the shortest growth rate (0.359, 0.327, 0.298, respectively) was obtained at 15 °C and 145 μmol photons m^−2^ s^−1^.

### 2.2. The Total Pigments Content for Three Phenotypes of the Genus Synechococcus

The acclimation mechanisms of three *Synechococcus* sp. phenotypes was characterized by the concentration of changes in composition and proportion of photosynthetic pigments i.e., chlorophyll *a* (Chl *a*), zeaxanthin (Zea), *β*-carotene (*β*-Car), phycoerythrin (PE), phycocyanin (PC), and allophycocyanin (APC) under different light (μmol photons m^−2^ s^−1^) and temperature (°C) conditions. In this work, the composition and proportions of Chl *a* and Car pigments (Zea and *β*-Car) of three *Synechococcus* sp. phenotypes were determined by HPLC method, while the content of phycobilins (Phyco) were determined by spectrophotometric method.

Both light and temperature significantly affected the cell-specific Chl *a* content of *Synechococcus* sp. Type 1, Type 2, and Type 3a (ANOVA, *p* < 0.001, for all) and Phyco content (ANOVA, *p* < 0.001, *p* < 0.001, and *p* < 0.001, for Type 1, Type 2, and Type 3a, respectively). Moreover, these factors significantly affected the cell-specific Car content of *Synechococcus* sp. phenotypes (ANOVA, *p* < 0.001, *p* < 0.001, *p* < 0.001 for Type 1, Type 2, and Type 3a, respectively; Table S2). Generally, ANOVA results indicated that the effect of irradiance on the Chl *a* and Phyco concentration for picocyanobacteria phenotypes was higher than the influence of temperature and the interaction of the two factors (Table S2). In contrast, the cell-specific Car content of *Synechococcus* sp. Type 1, Type 2, and Type 3a was more affected by temperature and the interaction of the two factors than by irradiance (Table S2).

**Figure 2:**
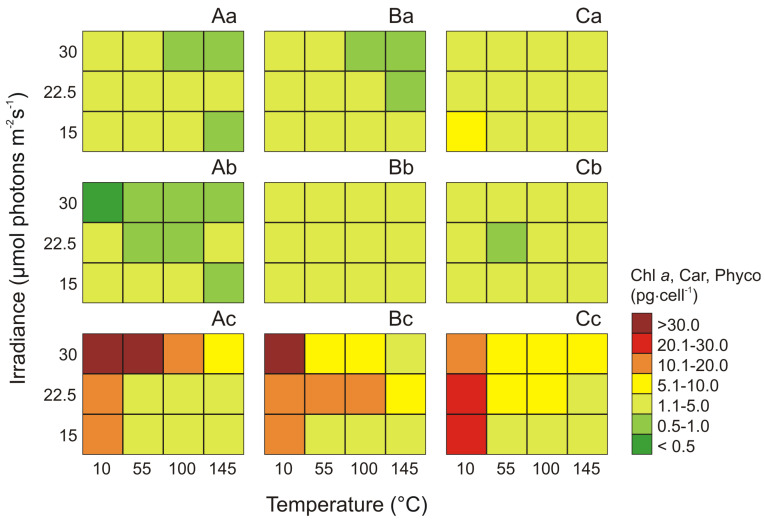
Changes in content (pg·cell^−1^) of Chl *a* (**a**), sum of total Car (**b**), and sum of total Phyco (**c**) obtained after 14 days of experiment for three phenotypes of *Synechococcus* sp.: Type 1 (**A**), Type 2 (**B**), Type 3a (**C**) under different irradiance (μmol photons m^−2^ s^−1^) and temperature (°C) conditions.

The maximum cell-specific concentration of Chl *a* (about 8.11 pg·cell^−1^) was noted for *Synechococcus* sp. Type 3a at 10 μmol photons m^−2^ s^−1^ light intensity and 15 °C, and it was about 5.5 times higher than the minimum at 145 μmol photons m^−2^ s^−1^ and 30 °C (Figure 2Ca). For *Synechococcus* sp. Type 1 and Type 2 the maximum cell-specific Chl *a* concentrations (4.51 pg·cell^−1^ and 4.82 pg·cell^−1^, respectively) were recorded at 10 μmol photons m^−2^ s^−1^ and 15 °C for Type 1 and 30 °C for Type 2. On the other hand, the minimum values for these phenotypes were obtained at 145 μmol photons m^−2^ s^−1^ and 30 °C (0.68 pg·cell^−1^ and 0.67 pg·cell^−1^, respectively; Figure 2Aa−Ba).

On the basis of the results obtained in this study, it was found that the analyzed phenotypes were characterized by a similar maximum cell-specific Car content. It was also shown that cell-specific Car content was the lowest among all analyzed photosynthetic pigments. The total Car content for *Synechococcus* sp. Type 1, Type 2, and Type 3a constituted approximately 7%, 11%, and 12% of the sum of Chl *a* and Phyco, respectively. It was also found that for Type 2 (Figure 2Bb) and Type 3a (Figure 2Cb) the maximum cell-specific Car content (2.01 pg·cell^−1^ and 2.25 pg·cell^−1^, respectively) were recorded at 190 μmol photons m^−2^ s^−1^ and 30 °C. By contrast, the minimum values of cell-specific Car content were obtained at 100 μmol photons m^−2^ s^−1^ and 22.5 °C (1.20 pg·cell^−1^, for Type 2 and 0.60 pg·cell^−1^, for Type 3a). On the other hand, for *Synechococcus* sp. Type 1, the reported maximum value of cell-specific Car content (1.74 pg·cell^−1^) at 100 μmol photons m^−2^ s^−1^ and 15 °C was approximately 4-fold higher compared to the recorded minimum values at 10 μmol photons m^−2^ s^−1^ and 30 °C (Figure 2Ab).

It was noted that the total Phyco pigments were always greater than cell-specific Chl *a* and Car content of the three examined *Synechococcus* sp. phenotypes. The study found that the total Phyco content for *Synechococcus* sp. Type 1, Type 2, and Type 3a constituted about 80%, 75%, and 65% of the sum of Chl *a* and Car, respectively. The highest cell-specific Phyco content was measured in *Synechococcus* sp. Type 2 (45.90 pg·cell^−1^) at 10 μmol photons m^−2^ s^−1^ and 30 °C (Figure 2Bc) while the minimum values of these pigments was noted at 55 μmol photons m^−2^ s^−1^ and 15 °C (2.70 pg·cell^−1^). The greatest decrease in the cell-specific Phyco content was noted for *Synechococcus* sp. Type 1 (Figure 2Ac), which under minimal conditions (100 μmol photons m^−2^ s^−1^ and 15 °C) was about 30 times lower than the recorded under maximum values at 10 μmol photons m^−2^ s^−1^ and 30 °C (33.56 pg·cell^−1^). In turn, *Synechococcus* sp. Type 3a showed the highest resistance to light and temperature, and its decrease in the cell-specific Phyco content under minimal conditions (145 μmol photons m^−2^ s^−1^ and 15 °C) was about 12.7 times lower (2.25 pg·cell^−1^) than the recorded under maximum values (10 μmol photons m^−2^ s^−1^ and 22.5 °C; Figure 2Cc).

### 2.3. Effect of Irradiance and Temperature on Phycocyanin, Phycoerythrin, and Allophycocyanin Content 

The presence of phycoerythrin (PE), phycocyanin (PC), and allophycocyanin (APC) was demonstrated for all picocyanobacterial phenotypes by spectrophotometric analysis. It was found that irradiance and temperature as well as their interaction significantly affected the cell-specific PE content of *Synechococcus* sp. (ANOVA, *p* < 0.001, for Type 1, Type 2, and Type 3a), PC content (ANOVA, *p* < 0.001, fot Type 1, *p* < 0.001, for Type 2, and *p* < 0.001, for Type 3a) and APC content (ANOVA, *p* < 0.001, *p* < 0.01, and *p* < 0.05, for Type 1, Type 2, and Type 3a, respectively; Table S3). ANOVA indicated that for most of *Synechococcus* sp. phenotypes, the effect of irradiance on PE was higher than the effect of temperature. In contrast, the PC and APC content of analyzed phenotypes was more affected by temperature than by irradiance and by the interaction of both factors (Table S3).

**Figure 3:**
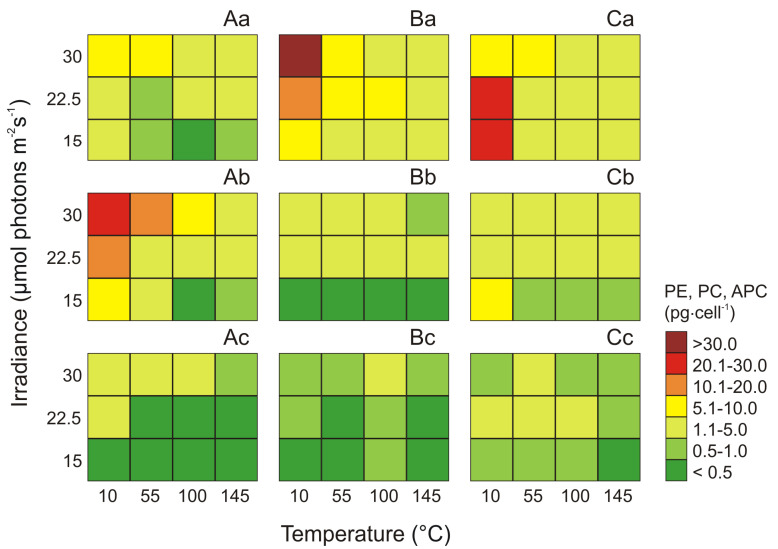
Changes in content (pg·cell^−1^) of PE (**a**), PC (**b**), and APC (**c**) obtained after 14 days of experiment for three phenotypes of *Synechococcus* sp.: Type 1 (**A**), Type 2 (**B**), Type 3a (**C**) under different irradiance (μmol photons m^−2^ s^−1^) and temperature (°C) conditions.

In all the phenotypes, the cell-specific (pg·cell^−1^) PE, PC, and APC pigment contents were environmentally driven (Figure 3). The cell-specific PE content increased with decrease of irradiance and increase of the temperature, reaching the highest values at the intensity of 10 μmol photons m^−2^ s^−1^ and temperature 22.5 °C (21.16 pg·cell^−1^ for Type 3a; Figure 3Ca) and 30 °C (8.59 pg·cell^−1^ for Type 1 and 40.35 pg·cell^−1^ for Type 2; Figure 3Aa,Ba). Under these conditions, the PE in the cells of the tested picocyanobacteria increased approximately 20.0-fold, 19.7-fold, and 13.6-fold, for Type 1, Type 2, and Type 3a, respectively, compared with the observed minimum values at 100–145 μmol photons m^−2^ s^−1^ and 15 °C.

On the basis of the conducted analyzes, it was found that the conditions under which the *Synechococcus* sp. Type 1 and Type 2 achieved the highest concentrations of the cell-specific PC were the low light intensity of 10 μmol photons m^−2^ s^−1^ and a high temperature of 30 °C. On the other hand, for Type 3a the maximal value of this pigment was noted at 10 μmol photons m^−2^ s^−1^ and 15 °C. The highest concentration value of PC pigments under optimal conditions was observed for *Synechococcus* Type 1 (20.95 pg·cell^−1^; Figure 3Ab), and the lowest for *Synechococcus* Type 2 (4.64 pg·cell^−1^; Figure 3Bb). The greatest decrease in cell-specific PC (about 64-fold) was noted for *Synechococcus* Type 1. However, the least susceptible to analyzed factors was *Synechococcus* Type 3a, with a 10-fold decrease in PC pigments (Figure 3Cb).

The highest cell-specific APC content (4.34 pg·cell^−1^) was recorded for *Synechococcus* sp. Type 1 in the 55 μmol photons m^−2^ s^−1^ and 30 °C (Figure 3Ac). For these light and temperature conditions, over 18-fold increase was observed in relation to the lowest recorded values at 10 μmol photons m^−2^ s^−1^ and 15 °C. For *Synechococcus* sp. Type 2 and Type 3a the maximum cell-specific APC concentrations (1.09 pg·cell^−1^ and 1.98 pg·cell^−1^, respectively) were recorded at 55−100 μmol photons m^−2^ s^−1^ and 22.5−30 °C. On the other hand, the minimum values for these phenotypes were obtained at 145 μmol photons m^−2^ s^−1^ and 15 °C (0.28 pg·cell^−1^ for Type 2 and 0.44 pg·cell^−1^, for Type 3a; Figure 3Bc,Cc). 

### 2.4. Effect of Irradiance and Temperature on Zeaxanthin and β-carotene

**Figure 4:**
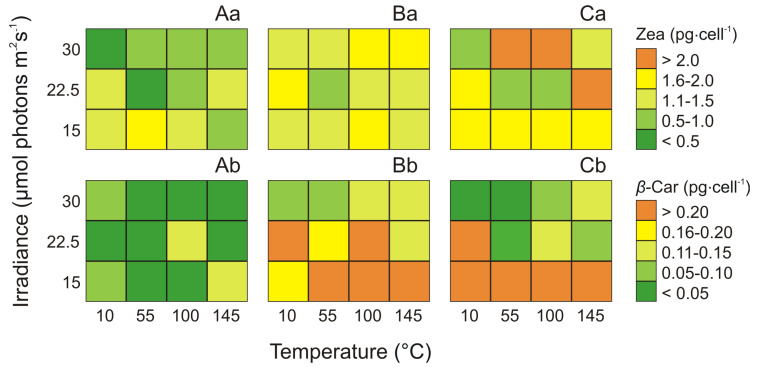
Changes in content (pg·cell^−1^) of Zea (**a**) and *β*-Car (**b**) obtained after 14 days of experiment for three phenotypes of *Synechococcus* sp.: Type 1 (**A**), Type 2 (**B**), Type 3a (**C**) under different irradiance (μmol photons m^−2^ s^−1^) and temperature (°C) conditions.

On the basis of the results, the effect of irradiance and temperature on changes in individual Car pigments in the cells of the picocyanobacterial phenotypes was determined. In all the *Synechococcus* sp. phenotypes, the cell-specific (pg·cell^−1^) pigment contents were environmentally driven (Figure 4). In the most of three tested phenotypes, the cell-specific concentrations of Zea (ANOVA, *p* < 0.001, *p* < 0.001, *p* < 0.001 for Type 1, Type 2, and Type 3a, respectively) and *β*-Car (ANOVA, *p* < 0.001, *p* < 0.01, *p* > 0.05 for Type 1, Type 2, and Type 3a, respectively) were affected by irradiance and temperature (Table S4). ANOVA indicated that in Type 1 and Type 3a, the effect of temperature on Zea was higher than the effect of irradiance. In contrast, the Zea content of Type 2 was more affected by irradiance than by temperature and by the interaction of both factors. It was also noted that for all tested phenotypes, effect of irradiance on *β*-Car was not statistically significant (Table S4).

The highest Zea content for *Synechococcus* sp. Type 2 and Type 3a (1.85 pg·cell^−1^ and 2.11 pg·cell^−1^, respectively) was noted at 100 μmol photons m^−2^ s^−1^ and 30 °C while the lowest value of this pigment were 1.02 pg·cell^−1^ for Type 2 and 0.53 pg·cell^−1^ for Type 3a at 55 μmol photons m^−2^ s^−1^ and 22.5 °C (Figure 4Ba,Ca). Moreover, the highest value of Zea content for Type 1 was found at irradiance 55 μmol photons m^−2^ s^−1^ and 15 °C (1.68 pg cell^−1^) while the minimum Zea content was obtained at 30 °C and 10 μmol photons m^−2^ s^−1^ (0.37 pg·cell^−1^; Figure 4Aa). The highest values of *β*-Car in Type 2 and Type 3a were noted at 55 μmol photons m^−2^ s^−1^ and 15 °C and 30 °C (0.32 pg·cell^−1^ and 0.40 pg·cell^−1^, respectively; Figure 4Bb,Cb). In turn, the lowest content of *β*-Car being found in Type 1 (0.12 pg·cell^−1^) at 145 μmol photons m^−2^ s^−1^ and 15 °C (Figure 4Ab). 

### 2.5. Effect of Irradiance and Temperature on Pigments Ratios

**Figure 5:**
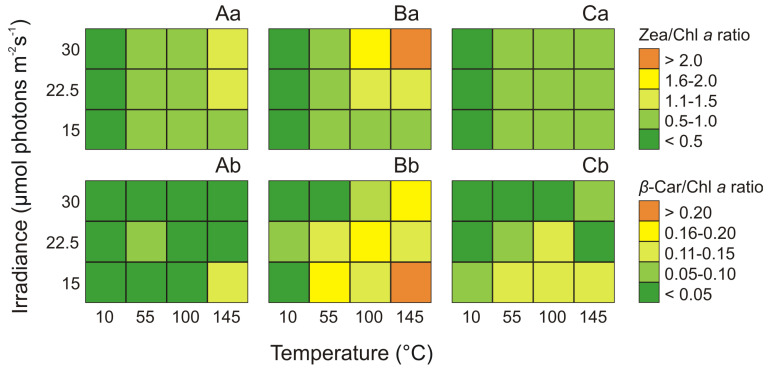
Changes in Zea/Chl *a* ratio (**a**) and *β*-Car/Chl *a* ratio (**b**) obtained after 14 days of experiment for three phenotypes of *Synechococcus* sp.: Type 1 (**A**), Type 2 (**B**), Type 3a (**C**) under different irradiance (μmol photons m^−2^ s^−1^) and temperature (°C) conditions.

Light and temperature as well as their interaction were found to significantly affect the Zea/Chl *a* ratio only in *Synechococcus* sp. Type 2 (ANOVA, *p* < 0.001) and the effect of light was higher than the effect of temperature and the interaction of both factors (Table S5). On the other hand, irradiance and temperature as well as their interaction significantly affected the *β*-Car/Chl *a* ratio in three *Synechococcus* sp. phenotypes (ANOVA, *p* < 0.001, *p* < 0.01, and *p* < 0.001, for Type 1, Type 2, and Type 3, respectively). ANOVA indicated that in Type 1 and Type 2, the effect of light on *β*-Car/Chl *a* ratio was higher than the effect of temperature. In contrast, the *β*-Car/Chl *a* ratio of Type 3a was more affected by temperature than by irradiance and by the interaction of both factors (Table S5). The highest values of Zea/Chl *a* ratio in *Synechococcus* sp. Type 2, at the 145 μmol photons m^−2^ s^−1^ and the temperature of 30 °C (2.3; Figure 5Ba) was about 11 times higher than the lowest values observed at the light intensity of 10 μmol photons m^−2^ s^−1^ and 30 °C. In turn, the lowest value of Zea/Chl *a* ratio was noted in Type 3a under the same light and temperature conditions (0.8; Figure 5Ca). Besides, the highest *β*-Car/Chl *a* ratio was also observed for *Synechococcus* sp. Type 2, which at the irradiance of 145 μmol photons m^−2^ s^−1^, and the temperature of 15 °C was 0.19 (Figure 5Bb). On the other hand, the lowest pigments ratio was recorded for *Synechococcus* sp. Type 3a, which under the same light and temperature conditions was 0.14 (Figure 5Cb).

Since Phyco pigments participate in the transfer of excitation energy to Chl *a* in photosystems, the analysis of changes in these pigments in relation to Chl *a* and Car was also performed (Table S6). It was found that irradiance and temperature as well as their interaction significantly affected the Phyco/Chl *a* ratio in *Synechococcus* sp. Type 1, Type 2, and Type 3 (ANOVA, *p* < 0.001, *p* < 0.001, and *p* < 0.01, respectively) and Phyco/Car ratio (ANOVA, *p* < 0.001, *p* < 0.001, and *p* < 0.001 for Type 1, Type 2, and Type 3a, respectively). ANOVA indicated that in Type 1 and Type 2, the effect of temperature on Phyco/Chl *a* ratio was higher than the effect of irradiance and the interaction of both factors. In turn, the Phyco/Chl *a* ratio of Type 3a was more affected by irradiance than by temperature. For Phyco/Car ratio the effect of temperature for three analyzed phenotypes was higher than the effect of irradiance and the interaction of both factors (Table S7).

The highest Phyco/Chl *a* ratio and Phyco/Car ratio were observed for *Synechococcus* sp. Type 1, which at the light intensity of 55 μmol photons m^−2^ s^−1^ and 10 μmol photons m^−2^ s^−1^ and the temperature of 30 °C was 16.5 and 62.5, respectively. Moreover, the highest values of these pigment ratio in Type 1 was about 33 times and 125 times, respectively higher than the lowest values observed at the light intensity of 100 μmol photons m^−2^ s^−1^ and 15 °C. Conversely, for *Synechococcus* sp. Type 3a the lowest values of Phyco/Chl *a* ratio as well as Phyco/Car ratio were found at 10 μmol photons m^−2^ s^−1^ and 22.5 °C (5.0 and 21.1, respectively) and were about 7 and 21 times higher, respectively, than the minimums obtained at PAR 100 μmol photons m^−2^ s^−1^ and 15 °C (Table S6).

## 3. Discussion

### 3.1. Occurrence and Abundance of Picocyanobacteria under Changing Irradiance and Temperature Conditions

Changes in the number of cells of photoautotrophic organisms inhabiting surface waters are the result of the interaction of several physical and chemical environmental factors [35]. Light and temperature play a key role in the occurrence of autotrophic picoplankton [32] and are the main factors causing the appearance of cyanobacteria both at depths and in coastal waters [36,37]. Additionally, light and temperature may be more important abiotic factors influencing the occurrence of picocyanobacteria than the availability of nutrients [36]. In spring, the number of autotrophic picoplankton cells begin to increase which is triggered by the temperature increase due to more intensive insolation of the surface water layers. Their growth reaches its maximum values during summer [36]. Gławdel et al. [38] showed that in the coastal waters of the southern Baltic Sea during the summer period, the autotrophic picoplankton, composed mainly of cyanobacteria in the total biomass exceeded even bacterioplankton. Three phenotypes of picocyanobacteria of the genus *Synechococcus* (Type 1, Type 2, and Type 3a) were isolated from the southern Baltic Sea. This area is characterized by large changes of environmental conditions. Autotrophic organisms living in such a variable ecosystem show the ability to quickly adapt which is essential for their survival. In this work, the influence of temperature and PAR irradiance on the autecology of the investigated phenotypes of *Synechococcus*: Type 1, Type 2, and Type 3a were demonstrated.

It was found that the increasing intensity of light had a negative effect on the cell concentration of the three studied phenotypes of *Synechococcus* sp. The number of picocyanobacteria cells increased as the PAR irradiance decreased, reaching the maximum value in the range of 10–55 μmol photons m^−2^ s^−1^ and the minimum value at 145 μmol photons m^−2^ s^−1^. Besides, it was shown that *Synechococcus* sp. Type 2 was the most susceptible to high light intensity. Its number of cells was more than 5-fold lower in high light compared to low light. On the other hand, the cell number decreased about 4-fold in the high light compared to low light for both *Synechococcus* sp. Type 1 and Type 3a. Literature data also indicated that picocyanobacteria of the genus *Synechococcus* in natural aquatic communities are adapted to low light and show maximum growth in the deeper layers of the euphotic zone [26,29,33,39,40]. The high abundance of autotrophic picoplankton was recorded even at a depth of 90 m [33]. This may indicate the ability of these organisms to survive seasonal changes and their fall into the aphotic zone. Besides, it is considered that *Synechococcus* sp. found in natural surface water layers may show photoinhibition of growth under high light [29,39,41] as well as the low rate of photosynthesis in the surface layer compared to greater depths [33,39]. On the other hand, Śliwińska-Wilczewska et al. [13] showed that the number of cells of green and brown phenotypes of *Synechococcus* sp. increased with the increase in light and was the highest in 280 μmol photons m^−2^ s^−1^. Furthermore, Kana and Glibert [42,43] showed that *Synechococcus* sp. could occur and grow in the irradiance reaching even 2000 μmol photons m^−2^ s^−1^. These studies confirmed that *Synechococcus* sp. can grow in maximally coastal waters due to their adaptation to high light intensities. Thus, picocyanobacteria of the genus *Synechococcus* can occur both at the near-surface layers and deeper waters. Furthermore, the ability of *Synechococcus* to grow in low light intensities and their low photoinhibition in exposure to high irradiance could give picocyanobacteria an advantage in changeable light-limited waters.

Temperature is also a very important factor controlling picocyanobacteria abundance in aquatic ecosystems [7,8,37]. Based on the conducted experiments, the influence of increasing temperature on the number of cells of the studied *Synechococcus* sp. phenotypes was found. The most favorable temperature conditions for the growth of *Synechococcus* sp. Type 1 and Type 2 were at 30 °C, while the highest number of cells for Type 3a was recorded at 22.5 °C. The most susceptible to high temperature was *Synechococcus* sp. Type 2. Its abundance was more than 5 times higher at 30 °C compared to the abundance recorded at 15 °C. On the other hand, for both *Synechococcus* sp. Type 1 and Type 3a, the increase in cell numbers along with the increase in temperature was about 4 times greater than that recorded at the lowest temperature. In laboratory studies, Jodłowska and Śliwińska [44] also found that increasing temperatures from 15 °C to 30 °C increased picocyanobacterial abundances. Similar observations were made by Śliwińska-Wilczewska et al. [13] who showed that with an increase in temperature from 10 °C to 25 °C, the number of cells of the green, red and brown *Synechococcus* sp. phenotype. was increased. Picocyanobacteria prefer high temperature for growth and their temperature optimum is higher than for eukaryotic phytoplankton organisms [37]. Furthermore, Paerl and Huisman [45] explained that the global temperature rise would stabilize or even inhibit the eukaryotic phytoplankton while the number of cyanobacteria would increase. Many cyanobacteria species demonstrate the highest increase in growth at 30−35 °C [46]. Noaman et al. [47] also demonstrated that the optimum temperature for growth of *Synechococcus leopoliensis* was 35 °C. An increase in temperature causes an increase in the number of picocyanobacteria cells, and their maximum occurrence was in the summer period when the water temperature is the highest [48]. This relationship is also apparent for the entire autotrophic picoplankton [49] and was confirmed by numerous studies [36,50,51]. Regarding climate change, picocyanobacteria of the genus *Synechococcus* achieves maximal growth rates at high temperatures and thus can be promoted by future global warming [7,8].

This study also showed that the analyzed phenotypes of *Synechococcus* sp.: Type 1, Type 2, and Type 3a has different growth rates. The highest growth rate was recorded for *Synechococcus* sp. Type 1. It was related to the smallest size obtained by these picocyanobacteria [44]. On the other hand, the lowest growth rate was observed for *Synechococcus* sp. Type 3a. Additionally, it was shown that this phenotype reached the largest cell size in cultures [44]. The research conducted by Stal et al. [52] on PE-rich and PC-rich phenotypes of *Synechococcus* also showed differences in the rate of cell growth depending on their size and picocyanobacteria with a larger cell size grew slower. Small cell size of *Synechococcus* Type 1 resulting in faster nutrient uptake allows picocyanobacteria to compete effectively with larger phytoplankton organisms in surface waters. On the other hand, increasing the cell volume of *Synechococcus* Type 3a may result in better light absorption at greater depths.

### 3.2. Changes in Pigments Content and Pigment Ratios under Different Irradiance and Temperature Conditions

Cyanobacteria living in coastal waters are often exposed to changes in light and temperature conditions. These factors influence the content of cyanobacterial photosynthetic pigments in aquatic ecosystems [53,54,55,56,57]. The factorial experiments performed in this study showed a negative effect of the increasing intensity of light on the cell-specific Chl *a* content for the three examined phenotypes of picocyanobacteria, obtaining the highest content at 10 μmol photons m^−2^ s^−1^ and the lowest for 145 μmol photons m^−2^ s^−1^. The conducted factorial experiments also showed a statistically significant influence of temperature on the cell-specific Chl *a* content for the examined phenotypes. The highest concentration of this pigment was observed at 30 °C for *Synechococcus* sp. Type 2 and at 15 °C for *Synechococcus* sp. Type 1 and Type 3a. The greatest decrease in the cell-specific Chl *a* content was noted for *Synechococcus* sp. Type 2, which under minimal conditions was about 7 times lower than the recorded under maximum values. On the other hand, *Synechococcus* sp. Type 3a showed the highest resistance to high values of irradiance, and its decrease in the content of Chl *a* in cells under minimal conditions was about 5.5 times higher than the recorded maximum values. Kana and Glibert [43] also showed that the concentration of this pigment was the highest for *Synechococcus* cells adapted to low light. On the other hand, the greatest decrease in Chl *a* content was recorded in the light greater than 700 μmol photons m^−2^ s^−1^ [42]. High content of Chl *a* in low light may indicate that picocyanobacteria of the genus *Synechococcus* may occur in highly shaded waters [52] and even under conditions of extreme radiation deficiency [58].

High light intensity is an unfavorable environmental factor for many photoautotrophic organisms [59]. However, cyanobacteria living in an environment with a high light intensity developed a defense strategy consisting of special pigmentation of the cells [39,60,61]. Convergence between the accumulation of Car pigments under the influence of high light intensity allows them to be assigned a protective role. The highest content of Zea and *β*-Car was recorded for *Synechococcus* sp. Type 3a. Zea is an accessory pigment at low light intensities but becomes dominant for cells growing under higher ones [16]. Our research showed that for the examined cyanobacteria cells the amount of Zea was much higher than that of *β*-Car. The study found that the Zea content for *Synechococcus* sp. Type 1, Type 2, and Type 3a was 93%, 89%, and 87% of the sum of Car pigments, respectively. Guillard et al. [62] observed that Zea may constitute as much as 50−81% of Car pigments for cyanobacteria of the genus *Synechococcus*. The high cell-specific Zea content in the *Synechococcus* sp. is related to the existence of these organisms in surface sea waters and places of exposure to high levels of solar radiation [62,63]. The cell-specific Car content of the tested picocyanobacteria phenotypes changed significantly in response to irradiance increase, which suggests that these organisms reorganize their pigments in order to protect against the unfavorable environmental conditions.

In this study, the factorial experiments carried out showed a negative effect of irradiance on the cell-specific PE, PC, and APC as well as the total sum of Phyco pigments content for the three studied phenotypes of the genus *Synechococcus*. Moreover, it was shown that the cell-specific content of these pigments increased with increasing temperature for Type 1 and Type 2. In turn, for Type 3a, a negative effect of increasing temperature on Phyco content was noted. On the basis of the conducted analyzes, it was found that the conditions under which the examined phenotypes of picocyanobacteria achieved the highest concentrations of the total sum of cell-specific Phyco content were at low light intensity of 10 μmol photons m^−2^ s^−1^ and high temperatures ranging between 22.5 and 30 °C. The greatest decrease in Phyco pigments (about 30-fold) in cyanobacteria cells under the influence of increasing light intensity was noted for *Synechococcus* Type 1. However, the least susceptible to high irradiance was *Synechococcus* Type 3a, with a 13-fold decrease in Phyco pigments. Among all Phyco pigments present in picocyanobacteria cells, the highest content of PE was observed for *Synechococcus* Type 2, whereas for *Synechococcus* Type 1 PC was the dominant pigment. A study by Kana and Glibert [42,43] also showed that the concentration of PE and PC were dependent on the intensity of light. The concentration of PC is related to the number of phycobilisomes [42]. The greatest increase of PC in cells was observed in low light, suggesting a change in phycobilisome numbers in growth-limiting light [42]. Cyanobacteria of the genus *Synechococcus*, depending on the light, can change their number and size of phycobilisomes and this may be associated with acclimatization to different light levels [42]. Photoaclimatization is visible when there is a reduction in photosynthetic pigments with increasing irradiance [64,65,66]. Hence, it may be concluded that the studied *Synechococcus* sp. phenotypes have a high ability to photoacclimatize to changing environmental conditions.

Based on conducted experiments, the highest Zea/Chl *a* ratio and *β*-Car/Chl *a* ratio was noted for *Synechococcus* sp. Type 2. On the other hand, the lowest ratios of the discussed pigments were recorded for *Synechococcus* sp. Type 3a. Tang and Vincent [67] showed that the content of Car and Chl *a* increases with increasing temperature. However, carotenoids grow more slowly with temperature, therefore the Car/Chl *a* ratio decreases with temperature [67]. Most cyanobacteria show photoinhibition at low temperatures [68], and an increase in the Car/Chl *a* ratio at low temperature may result in an increase in photoprotective pigments such as carotenoids [69,70]. Studies have shown that a high Car/Chl *a* ratio is characteristic for surface water populations [16]. In addition, Paerl et al. [71] and Paerl [72] suggested that a high Car/Chl *a* ratio has a dual role in cells as it maintains high photosynthetic light absorption capacity and protects cells from photooxidation which may explain why the deeper-living PE-rich *Synechococcus* sp. Type 2 had the highest Zea/Chl *a* ratio and *β*-Car/Chl *a* ratio of all studied phenotypes. This study also showed an increase in the Phyco/Chl *a* ratio and Phyco/Car ratio in the cells of the investigated cyanobacterial phenotypes with a decrease of irradiance and an increase of temperature. It is related to the advantage of Phyco pigments over Chl *a* and Car pigments for the tested picocyanobacteria phenotypes at low light intensity. Furthermore, a change in color from green, red and brown at low irradiances to bright yellow at high light levels was also observed for three phenotypes of cyanobacteria of the genus *Synechococcus* (Type 1, Type 2, and type 3a, respectively). A clear difference in the color of picocyanobacteria was associated with a change of the proportions between the pigments. At low light intensity, picocyanobacteria phenotypes showed the maximum content of Phyco and Chl *a* pigments. At the highest irradiance, the share of the Car pigments, mainly Zea, increased significantly in picocyanobacterial cells. Similar tendencies were observed by Kana and Glibert [16,42] for picocyanobacteria of the genus *Synechococcus*. Picocyanobacteria can acclimate to different light intensities by changing the content of pigments, especially Phyco and Chl *a* [73,74,75]. In this work, we observed the effect of light intensity and temperature on the cell-specific pigment content of all studied picocyanobacterial phenotypes. The concentration of Phyco and Chl *a* was the highest for picocyanobacteria cells acclimated to low light and decreased with increasing irradiance. Inverse relationships were noted for the cell-specific Car content. The high content of Phyco pigments and Chl *a* observed in our work indicated that the tested picocyanobacteria phenotypes are well adapted to low light conditions and high temperatures. Besides, the highest differences in the Phyco/Chl *a* ratio and Phyco/Car ratio were observed in *Synechococcus* sp. Type 1, which may confirm that this phenotype showed the best photoaclimatization abilities of all analyzed organisms. Because this PC-rich phenotype occurs in more productive waters [18,34,76], this observation may be important in the era of climate change and the associated mass occurrence of *Synechococcus* sp. in many places around the world [8,9]. It should be emphasized that Flombaum et al. [8] predicted that the number of *Synechococcus* sp. cells would increase by 14% at the end of the 21st century.

## 4. Materials and Methods

### 4.1. Culture Conditions

Three different phenotypes of picocyanobacteria from the genus *Synechococcus* were examined: BA-120 (Type 2), BA-124 (Type 1), and BA-132 (Type 3a). The strains were isolated from the coastal zone of the Gulf of Gdansk (the southern Baltic Sea) and maintained as unialgal cultures in the Culture Collection of Baltic Algae (CCBA) at the Institute of Oceanography, University of Gdańsk, Poland. Cyanobacteria were cultured on the BG-11 mineral medium [77], which was prepared with water from the Baltic Sea (salinity 8), which was filtered using 0.45 µm filters (Macherey-Nagel MN GF-5, Dueren, Germany) and autoclaved.

The cultures of cyanobacteria were acclimatized to the new conditions corresponding to the incubation conditions of the proper culture. After a week, the culture, which was in the logarithmic growth phase, was used to establish the proper, experimental culture. After the acclimatization time, proper cultures with known initial cell numbers were prepared. For this purpose, a specific volume of inoculum was taken from the actively growing acclimatization culture and added to the sterile media. The optimal number of the initial proper culture was set at 10^7^ cells in 1 mL of the medium. The inoculum selected in this way allowed for a constant increase in the number of cyanobacterial cells without inhibiting logarithmic population growth. The incubation of cultures lasted 14 days. After that time, for three phenotypes of cyanobacteria of the genus *Synechococcus* the cell concentration, the growth rate and photosynthetic pigments were determined. Each variant of the experiment was conducted in three repetitions and the results of the experiments were presented as an average of three measurements. 

The cultures of the examined cyanobacterial strains were carried out in thermostat under the following temperature conditions (°C): 15, 22.5, and 30. The effect of PAR irradiance was tested in photoperiod (16 h of light and 8 h of darkness) at the following values (μmol photons m^−2^ s^−1^): 10, 55, 100, and 145. 36 W Philips fluorescent lamps (Philips Lighting, Amsterdam, The Netherlands) were used as light sources and two additional 120 W halogen lamps by OSRAM (Osram Licht AG, Berlin, Germany) were used for the highest irradiance (145 μmol photons m^−2^ s^−1^). Measurements of PAR irradiance were made with Li-Cor (Lincoln, NE, USA), model LI-189 with cosine collector.

**Figure 6:**
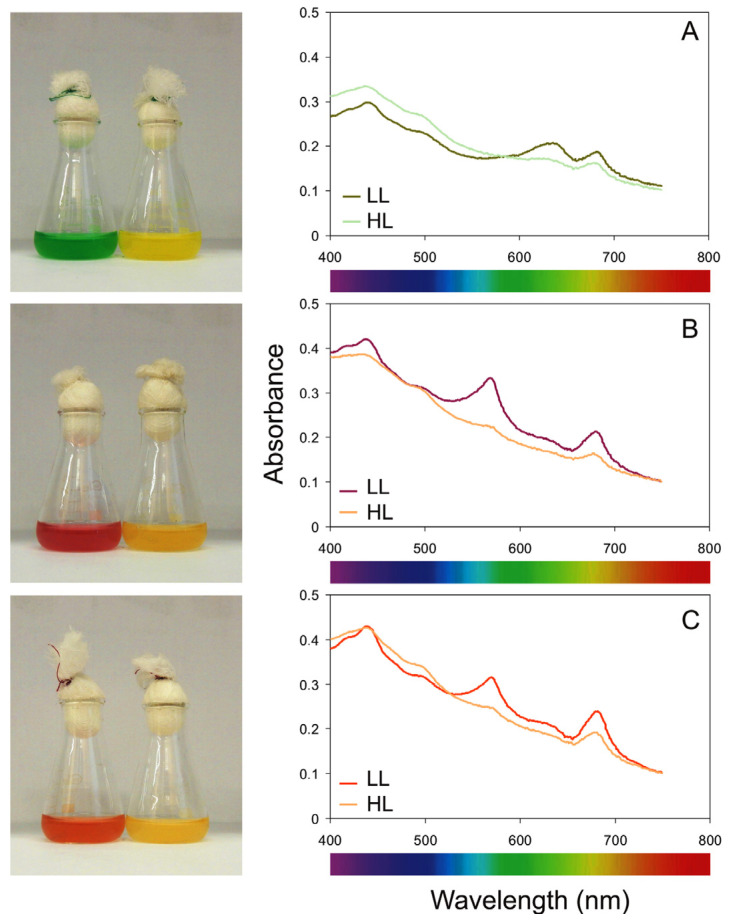
Left-side panel―photographs of the picocyanobacterial phenotypes in 100 mL glass Erlenmeyer flasks: Type 1 (**A**), Type 2 (**B**), and type 3a (**C**), obtained from low (left) and high (right) light; right-side panel―Absorbance spectra measured in the PAR range determined for the picocyanobacterial phenotypes at an optical density (OD_750_) = 0.1, obtained from low light (LL) and high light (HL).

It is worth mentioning here that a change in the color of the cultures of three phenotypes of picocyanobacteria *Synechococcus* sp. under different light was observed. The phenotypes were shown to be dark green, red and brown at low irradiance (for Type 1, Type 2, and type 3a, respectively), while in the high light their color turned to bright yellow. It was also shown that the examined phenotypes showed differences in PAR absorption spectra when exposed to low and high light (Figure 6).

### 4.2. Calculation of Cell Density and Growth Rates

Cell density was calculated using linear regression models based on cell concentration (N mL^−1^) and optical density (OD) at 750 nm [44]. Calculation of the cell number was conducted using the procedure described by Guillard and Sieracki [78], with a light microscope (Nikon Eclipse 80i, Nikon, Tokyo, Japan) and the Bürker counting chamber. To determine the growth rate of cyanobacteria, cell counts were conducted in cultures at two-day intervals from inoculation to the 14th day of culture. Based on these data the parameters characterizing the growth of cyanobacterial cells in the logarithmic phase: growth rate coefficient and cell doubling time were determined [78].

### 4.3. Determination of the Chlorophyll and Carotenoids Content

The concentration of photosynthetic pigments of analyzed picocyanobacteria was measured by the HPLC method. After 14 days of incubation, 40 mL of culture was filtered using 0.45 µm filters (Macherey-Nagel MN GF-5) to separate the picocyanobacteria cells from the medium. Chl *a* and Car were extracted from the picocyanobacteria cells with 90% acetone (V = 5 mL) and sonicated for 2 min. Then, the test-tube with the extract was held in the dark for 2 h at −80 °C. After 2 h, the pigment extract was centrifuged at 10,000 rpm for 5 min to remove filter particles (Sigma 2-16P, Osterode am Harz, Germany).

Chromatographic analyses were carried out using HPLC equipment of Waters company (Waters Chromatography Europe BV, Etten-Leur, The Netherlands) equipped with: Spectro Vision FD-300 fluorescence detector, Waters 486 absorption detector, Pharmacia autosampler LKB 2157, Waters Millennium Chromatography software. Measurements of pigment absorption were taken at 440 nm. Pigment separation was carried out according to a method proposed by Llewellyn and Mantour [79], with modifications [80] at room temperature on Vydac 201TP (C18) column 250 mm long. As an eluent A; 0.5 M ammonium acetate/methanol (20/80) was used and as eluent B; acetone/methanol (20/80) was used. Before injection of pigments extract (40 µL) the column was conditioned using an isocratic flow of eluents (40% A and 60% B) for 15 min. The analysis was performed at a flow rate of 1.0 mL min^−1^. Chl *a*, Zea, and *β*-Car standards were used for the qualitative and quantitative determination of pigments (The International Agency for 14C Determination, VKI, Hørsholm, Denmark). The pigments present in the cells of cyanobacteria strains of the genus *Synechococcus* were identified based on retention times and absorbance spectrum, which were compared with the standards. Calibration curves were plotted for each standard used to quantify assimilation pigments.

### 4.4. Determination of the Phycobiliproteins Content

The 40 mL of the test material was filtered through a 0.45 µm filter (Macherey-Nagel MN GF-5) and stored in −80 °C. Reagent for phycobiliprotein extraction contained 0.25 M Trizma Base, 10 mM binary EDTA and 2 mg mL^−1^ lysozyme. A pH of 5.5 was obtained by acidifying with concentrated HCl. The filters were homogenized in 5 mL of reagent, sonicated for 5 min and incubated first in the dark at 37 °C for about 2 h, then at 1.5 °C for about 20 h. After this time the pigment extract was centrifuged in experimental flasks for 10 min, at 10,000 rpm. Absorption measurements in 1 cm glass cuvettes on Beckman spectrophotometer (Indianapolis, IN, USA), model DU 530, at wavelengths (nm): 565, 620, 650 and 750, were conducted. The pigment contents: PE, PC, and APC were calculated based on Bennett and Bogorad [81] and Bryant et al. [82].

### 4.5. Statistical Analyses

To test the influence of a single factor as well as an interplay of factors on studied parameters the two-way ANOVA was used. Moreover, to determine the significance of treatment levels a post hoc test (Tukey’s HSD) was conducted. The impact of every environmental agent, as well as an interplay of factors on studied parameters, were measured using the method of orthogonal polynomial tables as described by Fisher and Yates [83]. Furthermore, to describe the connection of the factors and studied parameters regression equations were generated. Data are described as the mean ± standard deviation (SD). Levels of significance were * *p* < 0.05, ** *p* < 0.01, and *** *p* < 0.001. The statistical analyses were executed using the Statistica^®^ 13.1 software (StatSoft Polska, Cracow, Poland).

## 5. Conclusions

In this work, we found that the three analyzed phenotypes of the genus *Synechococcus* have diverse irradiance and temperature preferences. This, coupled with their high photoacclimation capabilities give them powerful tools to win the competition for the marine resources and provide them opportunity to dominate the area, at least as long as sufficient nutrient amounts are available. In almost all conditions the highest rate of growth was recorded for the *Synechococcus* sp. Type 1 which is the most competitive type. It prefers warmer waters −22.5 °C and above, but it produces the least nominal amounts of Car which is a probable cause of equalisation of the growth rates between the Type 1 and Type 2 at the highest irradiances and at the mentioned temperatures over 22 °C. The lowest growth rates were observed for the Type 3a for all variants. However, Type 3a was recognized to be less temperature sensitive and rather light-driven. Moreover, at low light and low temperature the highest pigment content was observed within the cells Type 3a which may suggest higher tolerance for colder waters such as tested here 15 °C or even below. The highest total pigment content per cell was recorded at 10 μmol photons m^−2^ s^−1^ at all temperature variants with the clear dominance of phycobilins among all the pigments. The high pigment content observed in picocyanobacteria cells proves that they may adapt and live in the deeper layers of the euphotic zone. The highest amounts of carotenoids were produced by Type 2. This may imply lower tolerance of this type to higher irradiance. Our results showed that the best photoaclimation abilities of all analyzed *Synechococcus* sp. types is Type 1 with the highest differences in the Phyco/Chl *a* and Phyco/Car ratios. One of our striking observations is a significant difference between the physiological responses of different *Synechococcus* sp. phenotypes to changeable environmental conditions. Thus, this work would be an important link in forecasting future changes in the occurrence of these organisms in the context of global warming.

## Supplementary Materials

The following are available online at https://www.mdpi.com/2073-4409/9/9/2030/s1, Table S1: Two-way factorial ANOVA of cells concentration measured in Synechococcus sp. Type 1, Type 2, and Type 3a growing at different temperatures (°C) and irradiance (μmol photons m^−2^ s^−1^): df—degrees of freedom; F—Fisher’s F-test statistic; Mss—mean sum of squares; Ss—sum of squares. Levels of significance were: * *p* < 0.05; ** *p* < 0.01; *** *p* < 0.001, Table S2: Two-way factorial ANOVA of cell-specific Chl *a*, Phyco, and Car content measured in *Synechococcus* sp. Type 1, Type 2, and Type 3a growing at different temperatures (°C) and irradiance (μmol photons m^−2^ s^−1^): df—degrees of freedom; F—Fisher’s F-test statistic; Mss—mean sum of squares; Ss—sum of squares. Levels of significance were: * *p* < 0.05; ** *p* < 0.01; *** *p* < 0.001, Table S3: Two-way factorial ANOVA of cell-specific PE, PC, and APC content measured in *Synechococcus* sp. Type 1, Type 2, and Type 3a growing at different temperatures (°C) and irradiance (μmol photons m^−2^ s^−1^): df—degrees of freedom; F—Fisher’s F-test statistic; Mss—mean sum of squares; Ss—sum of squares. Levels of significance were: * *p* < 0.05; ** *p* < 0.01; *** *p* < 0.001, Table S4: Two-way factorial ANOVA of cell-specific Zea and *β*-Car content measured in *Synechococcus* sp. Type 1, Type 2, and Type 3a growing at different temperatures (°C) and irradiance (μmol photons m^−2^ s^−1^): df—degrees of freedom; F—Fisher’s F-test statistic; Mss—mean sum of squares; Ss—sum of squares. Levels of significance were: * *p* < 0.05; ** *p* < 0.01; *** *p* < 0.001. Table S5: Two-way factorial ANOVA of Zea/Chl *a* ratio and *β*-Car/Chl *a* ratio measured in *Synechococcus* sp. Type 1, Type 2, and Type 3a growing at different temperatures (°C) and irradiance (μmol photons m^−2^ s^−1^): df—degrees of freedom; F—Fisher’s F-test statistic; Mss—mean sum of squares; Ss—sum of squares. Levels of significance were: * *p* < 0.05; ** *p* < 0.01; *** *p* < 0.001, Table S6: The Phyco/Chl *a* ratios and Phyco/Car ratios obtained after 14 days of experiment for three phenotypes of *Synechococcus* sp.: Type 1 (A), Type 2 (B), Type 3a (C) under different temperature (°C) and light (μmol photons m^−2^ s^−1^) conditions, Table S7: Two-way factorial ANOVA of Phyco/Chl *a* ratio and Phyco/Car ratio measured in *Synechococcus* sp. Type 1, Type 2, and Type 3a growing at different temperatures (°C) and irradiance (μmol photons m^−2^ s^−1^): df—degrees of freedom; F—Fisher’s F-test statistic; Mss—mean sum of squares; Ss—sum of squares. Levels of significance were: * *p* < 0.05; ** *p* < 0.01; *** *p* < 0.001.
