# Supplementary material for: Photosynthetic Pigments Changes of Three Phenotypes of Picocyanobacteria Synechococcus sp. under Different Light and Temperature Conditions
Source: Cells. 2020 Sep 3;9(9):2030. doi: 10.3390/cells9092030 (PMC7563753; doi:10.3390/cells9092030)
Supplement: Supplementary file 1 [file cells-09-02030-s001.pdf]

Supplementary material

Table S1. Two-way factorial ANOVA of cells concentration measured in *Synechococcus* sp. Type 1, Type 2, and Type 3a growing at different temperatures (°C) and irradiance (μmol photons $m^{-2}s^{-1}$): df – degrees of freedom; F – Fisher's F-test statistic; Mss – mean sum of squares; Ss – sum of squares. Levels of significance were: * $p < 0.05$; ** $p < 0.01$; *** $p < 0.001$.

Synechococcus sp.	Source of variation	Df	Ss	Mss	F
Type 1	temperature	2	39.464	19.732	118.1***
	irradiation	3	21.064	7.021	42.0***
	interaction	6	8.648	1.441	8.6***
	error	24	4.010	0.167	
Type 2	temperature	2	36.673	18.336	180.2***
	irradiation	3	10.130	3.377	33.2***
	interaction	6	2.393	0.399	3.9**
	error	24	2.442	0.102	
Type 3a	temperature	2	8.066	4.033	87.6***
	irradiation	3	4.690	1.563	34.0***
	interaction	6	1.363	0.227	4.9**
	error	24	1.105	0.046	

Table S2. Two-way factorial ANOVA of cell-specific Chl *a*, Phyco, and Car content measured in *Synechococcus* sp. Type 1, Type 2, and Type 3a growing at different temperatures (°C) and irradiance (μmol photons $m^{-2}s^{-1}$): df – degrees of freedom; F – Fisher's F-test statistic; Mss – mean sum of squares; Ss – sum of squares. Levels of significance were: * $p < 0.05$; ** $p < 0.01$; *** $p < 0.001$.

Synechococcus sp.	Cell-specific pigment content	Source of variation	Df	Ss	Mss	F
Type 1	Chl *a*	temperature	2	7.016	3.508	57.9***
		irradiation	3	20.639	6.880	113.6***
		interaction	6	8.683	1.447	23.9***
		error	24	1.453	0.061	
	Phyco	temperature	2	2108.089	1054.045	151.7***
		irradiation	3	1484.269	494.756	71.2***
		interaction	6	669.328	111.555	16.1***
		error	24	166.806	6.950	
	Car	temperature	2	2.800	1.400	208.1***
		irradiation	3	0.348	0.116	17.3***
		interaction	6	3.249	0.542	80.5***
		error	24	0.161	0.007	
Type 2	Chl *a*	temperature	2	0.544	0.272	9.0**
		irradiation	3	66.455	22.152	737.2***
		interaction	6	1.920	0.320	10.6***
		error	24	0.721	0.030	
	Phyco	temperature	2	713.744	356.872	96.8***
		irradiation	3	2523.931	841.310	228.3***
		interaction	6	1542.349	257.058	69.8***
		error	24	88.448	3.685	
	Car	temperature	2	0.218	0.109	3.8*
		irradiation	3	0.616	0.205	7.2**
		interaction	6	1.402	0.234	8.2***
		error	24	0.687	0.029	
Type 3a	Chl *a*	temperature	2	16.125	8.062	25.3***
		irradiation	3	46.366	15.455	48.5***
		interaction	6	32.352	5.392	16.9***
		error	24	7.642	0.318	
	Phyco	temperature	2	51.427	25.713	29.9***
		irradiation	3	2034.580	678.193	788.1***
		interaction	6	557.364	92.894	107.9***
		error	24	20.654	0.861	
	Car	temperature	2	2.749	1.374	8.7**
		irradiation	3	0.370	0.123	0.8
		interaction	6	7.481	1.247	7.9***
		error	24	3.795	0.158	

Table S3. Two-way factorial ANOVA of cell-specific PE, PC, and APC content measured in *Synechococcus* sp. Type 1, Type 2, and Type 3a growing at different temperatures (°C) and irradiance (μmol photons $m^{-2}s^{-1}$): df – degrees of freedom; F – Fisher's F-test statistic; Mss – mean sum of squares; Ss – sum of squares. Levels of significance were: * $p < 0.05$; ** $p < 0.01$; *** $p < 0.001$.

Synechococcus sp.	Cell-specific pigment content	Source of variation	Df	Ss	Mss	F
Type 1	PE	temperature	2	134.884	67.442	116.1***
		irradiation	3	89.066	29.689	51.1***
		interaction	6	39.980	6.663	11.5***
		error	24	13.940	0.581	
	PC	temperature	2	736.334	368.167	99.0***
		irradiation	3	602.840	200.947	54.0***
		interaction	6	294.321	49.054	13.2***
		error	24	89.276	3.720	
	APC	temperature	2	37.955	18.978	108.2***
		irradiation	3	13.442	4.481	25.6***
		interaction	6	18.304	3.051	17.4***
		error	24	4.209	0.175	
Type 2	PE	temperature	2	499.665	249.833	126.7***
		irradiation	3	2236.924	745.641	378.2***
		interaction	6	1189.413	198.235	100.6***
		error	24	47.311	1.971	
	PC	temperature	2	13.915	6.957	52.0***
		irradiation	3	9.946	3.315	24.8***
		interaction	6	20.718	3.453	25.8***
		error	24	3.210	0.134	
	APC	temperature	2	1.012	0.506	63.0***
		irradiation	3	1.112	0.371	46.1***
		interaction	6	0.251	0.042	5.2**
		error	24	0.193	0.008	
Type 3a	PE	temperature	2	42.136	21.068	22.4***
		irradiation	3	1374.341	458.114	486.6***
		interaction	6	311.636	51.939	55.2***
		error	24	22.593	0.941	
	PC	temperature	2	9.746	4.873	70.0***
		irradiation	3	45.038	15.013	215.5***
		interaction	6	32.867	5.478	78.6***
		error	24	1.672	0.070	
	APC	temperature	2	4.543	2.272	34.2***
		irradiation	3	3.813	1.271	19.1***
		interaction	6	1.304	0.217	3.3*
		error	24	1.593	0.066	

Table S4. Two-way factorial ANOVA of cell-specific Zea and *β*-Car content measured in *Synechococcus* sp. Type 1, Type 2, and Type 3a growing at different temperatures (°C) and irradiance (μmol photons $m^{-2}s^{-1}$): df – degrees of freedom; F – Fisher's F-test statistic; Mss – mean sum of squares; Ss – sum of squares. Levels of significance were: * $p < 0.05$; ** $p < 0.01$; *** $p < 0.001$.

Synechococcus sp.	Cell-specific pigment content	Source of variation	Df	Ss	Mss	F
Type 1	Zea	temperature	2	2.511	1.255	236.3***
		irradiation	3	0.272	0.091	17.0***
		interaction	6	3.451	0.575	108.3***
		error	24	0.127	0.005	
	β-Car	temperature	2	0.002	0.001	1.2
		irradiation	3	0.004	0.001	2.2
		interaction	6	0.034	0.006	8.9***
		error	24	0.015	0.001	
Type 2	Zea	temperature	2	0.086	0.043	3.4*
		irradiation	3	0.788	0.263	20.9***
		interaction	6	0.743	0.124	9.9***
		error	24	0.301	0.013	
	β-Car	temperature	2	0.153	0.077	29.6***
		irradiation	3	0.006	0.002	0.7
		interaction	6	0.067	0.011	4.3**
		error	24	0.062	0.003	
Type 3a	Zea	temperature	2	1.155	0.577	6.2**
		irradiation	3	0.445	0.148	1.6
		interaction	6	6.646	1.108	11.9***
		error	24	2.242	0.093	
	β-Car	temperature	2	0.644	0.322	52.6***
		irradiation	3	0.035	0.012	1.9
		interaction	6	0.074	0.012	2.0
		error	24	0.147	0.006	

Table S5. Two-way factorial ANOVA of Zea/Chl *a* ratio and *β*-Car/Chl *a* ratio measured in *Synechococcus* sp. Type 1, Type 2, and Type 3a growing at different temperatures (°C) and irradiance (μmol photons $m^{-2}s^{-1}$): df – degrees of freedom; F – Fisher's F-test statistic; Mss – mean sum of squares; Ss – sum of squares. Levels of significance were: * $p < 0.05$; ** $p < 0.01$; *** $p < 0.001$.

Synechococcus sp.	Cell-specific pigment ratio	Source of variation	Df	Ss	Mss	F
Type 1	Zea/Chl *a*	temperature	2	0.010	0.005	0.3
		irradiation	3	4.097	1.366	73.9***
		interaction	6	0.243	0.041	2.2
		error	24	0.444	0.018	
	β-Car/Chl *a*	temperature	2	0.001	0.001	2.1
		irradiation	3	0.017	0.006	21.1***
		interaction	6	0.031	0.005	18.7***
		error	24	0.007	0.000	
Type 2	Zea/Chl *a*	temperature	2	1.564	0.782	100.4***
		irradiation	3	9.929	3.310	425.2***
		interaction	6	2.307	0.384	49.4***
		error	24	0.187	0.008	
	β-Car/Chl *a*	temperature	2	0.019	0.009	6.0**
		irradiation	3	0.082	0.027	17.5***
		interaction	6	0.037	0.006	3.9**
		error	24	0.038	0.002	
Type 3a	Zea/Chl *a*	temperature	2	0.015	0.008	2.2
		irradiation	3	1.467	0.489	141.5***
		interaction	6	0.049	0.008	2.4
		error	24	0.083	0.003	
	β-Car/Chl *a*	temperature	2	0.037	0.019	117.3***
		irradiation	3	0.022	0.007	46.2***
		interaction	6	0.021	0.003	21.6***
		error	24	0.004	0.000	

Table S6. The Phyco/Chl *a* ratios and Phyco/Car ratios obtained after 14 days of experiment for three phenotypes of *Synechococcus* sp.: Type 1 (A), Type 2 (B), Type 3a (C) under different temperature (°C) and light (μmol photons $m^{-2}s^{-1}$) conditions.

**Experimental conditions: temperature (°C); irradiance (μmol photons $m^{-2}s^{-1}$)**	Phyco/Chl *a*			Phyco/Car		
	A	B	C	A	B	C
15; 10	2.3±0.3	2.4±0.6	3.5±0.3	6.4±0.8	6.3±1.3	9.6±1.4
15; 55	1.0±0.1	1.4±0.2	1.8±0.7	1.6±0.2	1.4±0.3	2.5±1.1
15; 100	0.5±0.1	1.9±0.7	0.7±0.1	0.5±0.1	1.9±0.7	1.0±0.0
15; 145	2.1±0.6	1.9±0.6	1.0±0.4	1.6±0.5	1.8±0.6	1.0±0.4
22.5; 10	6.9±2.0	4.5±1.5	5.0±0.5	25.6±0.8	15.6±3.5	21.1±1.3
22.5; 55	1.7±0.1	7.2±2.3	4.2±3.5	2.4±0.1	8.2±1.3	5.9±4.5
22.5; 100	2.6±1.4	7.6±2.1	2.2±0.4	3.3±1.9	5.7±1.3	2.9±0.6
22.5; 145	3.4±2.2	4.6±0.9	1.5±0.6	4.0±2.7	3.8±1.5	1.7±0.6
30; 10	13.1±2.1	8.7±1.0	2.1±0.9	62.5±5.3	27.8±0.3	7.8±3.4
30; 55	16.5±1.9	3.3±0.1	2.6±0.4	35.4±2.8	5.5±2.3	4.3±0.8
30; 100	12.2±0.0	4.7±0.4	1.5±0.4	12.4±1.3	2.8±0.2	2.1±0.6
30; 145	10.2±0.7	3.5±0.1	5.0±0.5	8.8±0.6	2.1±0.6	4.3±0.2

Table S7. Two-way factorial ANOVA of Phyco/Chl *a* ratio and Phyco/Car ratio measured in *Synechococcus* sp. Type 1, Type 2, and Type 3a growing at different temperatures (°C) and irradiance (μmol photons $m^{-2}s^{-1}$): df – degrees of freedom; F – Fisher's F-test statistic; Mss – mean sum of squares; Ss – sum of squares. Levels of significance were: * $p < 0.05$; ** $p < 0.01$; *** $p < 0.001$.

Synechococcus sp.	Cell-specific pigment ratio	Source of variation	Df	Ss	Mss	F
Type 1	Phyco/Chl *a*	temperature	2	899.094	449.547	273.9***
		irradiation	3	32.637	10.879	6.6**
		interaction	6	80.841	13.474	8.2***
		error	24	39.395	1.641	
	Phyco/Car	temperature	2	4884.169	2442.085	586.1***
		irradiation	3	4181.679	1393.893	334.5***
		interaction	6	2533.782	422.297	101.4***
		error	24	99.998	4.167	
Type 2	Phyco/Chl *a*	temperature	2	111.229	55.614	45.2***
		irradiation	3	18.543	6.181	5.0**
		interaction	6	65.828	10.971	8.9***
		error	24	29.502	1.229	
	Phyco/Car	temperature	2	306.250	153.125	70.0***
		irradiation	3	1150.132	383.377	175.2***
		interaction	6	495.006	82.501	37.7***
		error	24	52.521	2.188	
Type 3a	Phyco/Chl *a*	temperature	2	13.711	6.855	5.4*
		irradiation	3	19.799	6.600	5.2**
		interaction	6	39.000	6.500	5.2**
		error	24	30.289	1.262	
	Phyco/Car	temperature	2	125.716	62.858	19.4***
		irradiation	3	701.862	233.954	72.1***
		interaction	6	227.182	37.864	11.7***
		error	24	77.912	3.246	
